# Clinical Significance of Incidentally Detected Parotid Masses on Brain MRI and PET-CT

**DOI:** 10.3390/diagnostics15222895

**Published:** 2025-11-14

**Authors:** Joong Seob Lee, Jeong In Jang, Jee Hye Wee, Jeong Wook Kang, Ho Suk Kang, Mi Jung Kwon, Heejin Kim

**Affiliations:** 1 Department of Otorhinolaryngology-Head and Neck Surgery, Hallym University Sacred Heart Hospital, College of Medicine, Hallym University, Anyang 14068, Republic of Korea; apniosio@naver.com (J.S.L.); 230182@hallym.or.kr (J.I.J.); weejh07@hanmail.net (J.H.W.); simbody@naver.com (J.W.K.); 2 Division of Gastroenterology, Department of Internal Medicine, Hallym University Sacred Heart Hospital, College of Medicine, Hallym University, Anyang 14068, Republic of Korea; hskang76@hallym.or.kr; 3 Department of Pathology, Hallym University Sacred Heart Hospital, College of Medicine, Hallym University, Anyang 14068, Republic of Korea; mulank99@hallym.or.kr

**Keywords:** parotid tumor, brain MRI, PET-CT

## Abstract

**Background/Objectives**: Parotid incidentalomas are increasingly detected during brain MRI and PET-CT, particularly in patients with serious diseases such as cancer. This study aimed to evaluate the clinical significance of incidentally identified parotid lesions. **Methods**: We retrospectively reviewed the records of 44,952 patients (≥19 years) who underwent brain MRI and 10,957 who underwent PET-CT between January 2014 and December 2023. The incidence, imaging findings, and pathological results of parotid incidentalomas were analyzed. **Results**: Among 44,952 brain MRIs, 100 incidental parotid lesions (0.22%) were detected, compared with 92 lesions (0.84%) among 10,957 PET-CT scans. The mean patient age was slightly higher in the PET-CT group. Of the MRI-detected lesions, 35 patients underwent further evaluation and 14 underwent surgery, with final pathology confirming only benign tumors, including pleomorphic adenomas, Warthin tumors, and basal cell adenomas. In contrast, among 23 PET-CT patients who underwent additional evaluation, 7 had surgery, and final pathology revealed both benign and malignant tumors. Malignant cases included mucoepidermoid carcinoma, metastatic Merkel cell carcinoma, metastatic sebaceous carcinoma, and adenoid cystic carcinoma. Notably, two patients with initially benign cytology and negative PET-CT findings were later confirmed to have malignancies after surgery, Primary sites of metastatic disease included the thyroid, cervix, head and neck, and skin. **Conclusions**: Most parotid incidentalomas detected on brain MRI are benign and may be managed conservatively. However, incidentalomas identified on PET-CT require thorough evaluation, as they may indicate metastatic disease or a second primary malignancy, particularly in patients with head and neck or skin cancers.

## 1. Introduction

Most parotid neoplasms can be readily detected through palpation or visual inspection. However, some are incidentally discovered during unrelated imaging studies, such as brain magnetic resonance imaging (MRI) or 18-F fluorodeoxyglucose (FDG) positron emission tomography-computed tomography (PET-CT)—particularly in patients with short necks or deeply located parotid lesions. Although parotid gland tumors are relatively uncommon, approximately 75% are benign [1,2,3]. Among these, pleomorphic adenoma is the most frequent benign tumor, with a reported risk of malignant transformation ranging from 1.8 to 6.2% [4,5].

For preoperative biopsy, fine-needle aspiration cytology (FNAC) is commonly performed as an initial diagnostic tool for salivary gland masses because it is relatively safe and easy to perform. However, due to the histologic heterogeneity of salivary gland tumors, the reported sensitivity of FNAC has been inconsistent, ranging from 48% to 94% for subclassifying neoplasms [6,7,8,9]. Owing to this variability and the relatively low diagnostic accuracy of preoperative biopsy, complete surgical excision is generally recommended to establish a definite diagnosis.

18 F-FDG PET-CT is a widely used imaging modality for cancer staging, particularly for detecting distant metastases and identifying occult primary malignancies. Parotid incidentalomas are frequently reported on PET-CT, with a prevalence ranging from 0.3% to 0.4% [10,11]. In one series, 558 incidentalomas were identified, of which 256 were benign and 112 were malignant [12]. Because small deep-lobe parotid tumors may not be palpable, they are often detected incidentally. A meta-analysis further demonstrated that deep-lobe parotid tumors have a malignancy rate of 26.6%, which is higher than that of tumors located in the superficial lobe [13].

Brain MRI is another commonly used imaging modality, often performed to evaluate conditions such as brain tumors, stroke, chronic headache, or facial nerve palsy. Many patients undergoing brain MRI or PET-CT as part of cancer or stroke evaluations are in poor general condition and are already receiving treatment for their primary disease. Consequently, determining whether to pursue further evaluation or treatment for an incidentally discovered parotid lesion can be clinically challenging.

In this study, we reviewed the clinical courses of patients with parotid incidentalomas detected on brain MRI or PET-CT performed during evaluations for their primary conditions and assessed the clinical relevance of these findings.

## 2. Materials and Methods

### 2.1. Patients

This retrospective study included 10,957 adult patients (aged ≥ 19 years) who underwent PET-CT and 44,952 patients who underwent brain MRI at our hospital between January 2014 and December 2023. The study was approved by the Institutional Review Board (IRB) of our hospital (IRB No. 2023-12-015-001) on 5 March 2024.

To identify potential parotid incidentalomas, PET-CT and brain MRI reports were screened for the keywords parotid, parotid gland, or PG. This search yielded 231 cases from PET-CT reports and 140 from MRI reports. Patient images and clinical histories were reviewed using the electronic medical record (EMR) system and the picture archiving and communication system (PACS).

Patients with lymphoproliferative diseases or radiologic findings suggestive of multiple lymph node metastases—where a parotid lesion could represent lymphoproliferative involvement or intra-parotid lymph node metastasis without further diagnostic work-up—were excluded. Additionally, patients with a prior diagnosis or treatment of parotid malignancy were also excluded.

**Figure 1:**
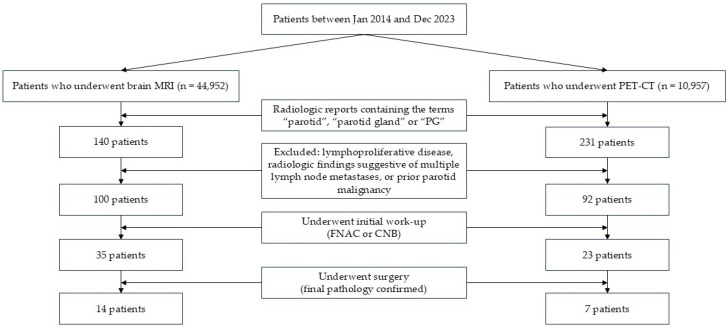
Flowchart of patients with parotid incidentalomas who underwent brain magnetic resonance imaging (MRI) and positron emission-computed tomography (PET-CT).

After applying these exclusion criteria, 92 PET-CT cases and 100 brain MRI cases with parotid incidentalomas were included in the analysis, as illustrated in the flowchart (Figure 1).

Lesion size was measured, and radiologic interpretations were reviewed. From the EMR, we also extracted data on the primary indication for imaging (brain MRI or PET-CT), biopsy results—including FNAC and core needle biopsy (CNB)—and each patient’s Eastern Cooperative Oncology Group (ECOG) performance status [14] and American Society of Anesthesiologists (ASA) physical status classification [15].

The final study population comprised 192 patients: 115 men (59.9%) and 77 women (40.1%), with ages ranging from 19 to 92 years (mean age, 68.7 ± 12.6 years). Of these, 100 patients underwent brain MRI (47 men and 53 women; mean age, 68.90 ± 12.61 years) and 92 underwent PET-CT (68 men and 24 women; mean age, 69.49 ± 11.64 years) (Table 1). Detailed clinical characteristics are summarized in Table 1.

### 2.2. Statistical Analysis

Statistical analyses were performed using IBM SPSS Statistics software (version 27.0; IBM Corp., Armonk, NY, USA). The prevalence of incidental parotid masses and individual disease entities was evaluated. Continuous variables were expressed as mean ± standard deviation (SD). Student’s *t*-test and the chi-square test were used to analyze patient demographic data. A *p*-value < 0.05 was considered statistically significant for all analyses.

## 3. Results

### 3.1. Incidence of Parotid Incidentalomas on Brain MRI and PET-CT

Among the 44,952 brain MRI scans, 100 parotid lesions (0.22%) were incidentally detected. In comparison, 92 parotid lesions (0.84%) were identified among 10,957 PET-CT scans. The mean age was slightly higher in the PET-CT group than in the MRI group (69.49 ± 11.64 vs. 68.90 ± 12.61 years, respectively). Among the MRI-detected lesions, 35 patients (35.0%) underwent further evaluation, such as FNAC or CNB (Table 1). In the PET-CT group, 23 patients underwent additional evaluation for parotid incidentalomas. Of these, 14 patients in the MRI group and 7 in the PET-CT group underwent surgical excision for their parotid lesions.

Table 2 summarizes the indications for PET-CT scans in patients with parotid incidentalomas and the stages of their primary cancers. Lung cancer was the most common primary malignancy associated with parotid incidentalomas. Head and neck cancers and skin cancers were also relatively frequent, despite their lower overall incidence. Advanced-stage primary cancers were identified in approximately half of the patients with parotid incidentalomas.

### 3.2. Characteristics of Patients with Parotid Incidentalomas Who Underwent Further Evaluation

In the MRI group, the mean age did not differ significantly between patients who underwent further evaluation and those who did not (69.32 ± 9.96 years vs. 68.65 ± 14.01 years, *p* = 0.182) (Table 3). The mean size of the parotid mass was slightly larger in patients who underwent additional evaluation (1.87 ± 0.61 vs. 1.83 ± 0.99 cm, *p* = 0.067), although the difference was not statistically significant. General patient conditions, including ECOG performance status and ASA physical status classification, were also comparable between the two groups. The clinical indications for MRI among patients who underwent further evaluation were diverse, ranging from mild to severe conditions such as stroke, cancer work-up, neuropathy, and dizziness.

In contrast, a smaller proportion of patients (23 patients, 25.0%) in the PET-CT group underwent further evaluation for parotid lesions (Table 4). Similar to the MRI group, there were no significant differences in sex or mean age between those who did and did not undergo additional evaluation. The mean SUV was slightly higher in patients who underwent further evaluation, but the difference was not statistically significant (7.02 ± 2.97 vs. 6.77 ± 2.73, *p* = 0.859). Interestingly, the stages of primary cancer were relatively lower in patients who underwent additional evaluation, and many had primary head and neck (laryngeal, tonsillar, and nasopharyngeal) or thyroid cancers.

### 3.3. Comparison Between Initial Work-Up and Final Pathologic Results

**Figure 2:**
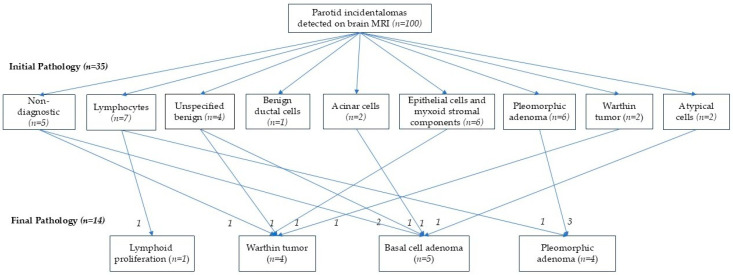
Pathologic results of patients with parotid incidentalomas detected on brain MRI, comparing initial pathology from fine-needle aspiration cytology or core needle biopsy with final pathology from surgical resection.

Figure 2 presents the histologic results of parotid incidentalomas detected on brain MRI. Initial cytologic diagnoses obtained through FNAC or CNB included 4 cases of unspecified benign lesions, 6 with epithelial cells and myxoid stromal components, 1 with benign ductal cells, 7 with lymphocytes, 2 with acinar cells, 5 with cystic or fibrotic exudate or scanty cellularity (non-diagnostic), 2 with atypical cells, 6 pleomorphic adenomas, and 2 Warthin tumors. Among these, 14 patients underwent surgical resection of their incidentally detected parotid lesions. Final pathology confirmed 4 pleomorphic adenomas, 4 Warthin tumors, 5 basal cell adenomas, and 1 case of lymphoid proliferation—all classified as benign tumors (Figure 2). All MRI interpretations in surgically treated patients were also reported as benign.

**Figure 3:**
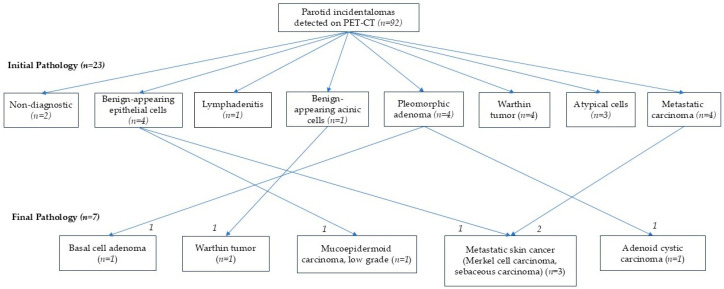
Pathologic results of patients with parotid incidentalomas detected on PET-CT, comparing initial fine-needle aspiration cytology (FNAC) or core needle biopsy (CNB) with final pathology from surgical resection.

In the PET-CT group, the initial diagnoses were as follows: 4 cases of benign-appearing epithelial cells, 1 of benign-appearing acinic cells, 1 lymphadenitis, 4 metastatic carcinomas, 2 with cystic changes or scant cellularity (non-diagnostic), 3 with atypical cells, 4 pleomorphic adenomas, and 4 Warthin tumors (Figure 3). Among these patients, 7 underwent surgery. Final pathological diagnoses included 1 mucoepidermoid carcinoma (low grade), 1 metastatic Merkel cell carcinoma, 1 metastatic carcinoma, 1 metastatic sebaceous carcinoma, 1 adenoid cystic carcinoma, and 1 basal cell adenoma. Notably, in two cases, the initial pathology showed benign-appearing epithelial cells, and the PET-CT report did not suggest malignancy; however, malignancy was confirmed after surgical excision. Additionally, one case initially diagnosed as pleomorphic adenoma was later confirmed as adenoid cystic carcinoma. In the case initially diagnosed as metastatic carcinoma, metastasis was also confirmed in the final pathology. The primary sites in patients with initially benign but ultimately malignant parotid lesions were the thyroid and cervix, while four other metastatic cases originated from primary cancers of the skin and head and neck region.

### 3.4. Clinical Outcomes During Follow-Up of Parotid Incidentalomas

The median follow-up period was 14.5 month (range, 1–148 months) in the PET-CT group, with 35% of patients followed for less than 6 months. In the MRI group, the median follow-up period was 16 months (range, 1–120 months). Twenty patients in the PET-CT group died from their primary diseases during follow-up. In the MRI group, 14 patients died from their primary disease, and 19 underwent follow-up imaging without surgery. Of these, 17 showed stable lesion size, one lesion increased, and one resolved spontaneously. Among the PET-CT group, 19 patients underwent follow-up imaging without surgery; 15 showed no change in size, 4 showed increased size, and 1 showed decreased size. No patients in either group died of parotid disease.

## 4. Discussion

With advances in radiological imaging, PET-CT and MRI have been increasingly utilized in diverse clinical settings. PET-CT offers whole-body coverage, and many parotid tumors demonstrate high SUV uptake regardless of whether they are malignant or benign [16]. Consequently, parotid incidentalomas are frequently detected and have been increasingly reported. A recent systematic review of 558 parotid incidentalomas found a mean incidence of 0.74% on PET-CT, which is comparable to our finding (0.84%) [12]. Another large study reported a higher incidence (1.73%), likely due to the inclusion of non-focal ultrasonographic findings and metastatic lesions from lymphoma [17]. In contrast, our study excluded patients with lymphoproliferative disease, parotid gland metastases in interpretation, or a history of parotid malignancy to better define true parotid incidentalomas, which may explain the lower incidence observed.

The most common indication for PET-CT in both previous studies [12,17] and in our series was lung cancer. Among parotid incidentalomas, Warthin tumor is one of the most frequent findings on PET-CT because of its high glucose avidity [18,19]. A recent meta-analysis of PET-CT–detected parotid incidentalomas involving Warthin tumor also demonstrated a low referral rate for further evaluation and a similar median SUV uptake (7.21) [20]. However, that study was limited by a lack of detailed histopathologic data, as a surgical confirmation was performed in only three cases. Consistent with prior reports, our series also showed a predominance of benign lesions among parotid incidentalomas, although malignant lesions were observed in PET-CT–detected group [12,17].

Regarding MRI, a previous study of 7262 patients reported a 1.2% incidence of parotid incidentalomas (86/7292), which was higher than that in our study (0.22%) [21]. Pathologic evaluation was performed in 49 of those 86 cases (57.0%), with most lesions (46/49, 93.9%) being benign—consistent with our findings. In contrast, while no malignant cases were identified in our MRI group, their study reported three malignant parotid incidentalomas (one mucoepidermoid carcinoma and two metastatic lymph nodes), likely reflecting inclusion of metastatic lymph node lesions within the parotid region.

Benign parotid tumors typically present as slowly growing, painless masses and may warrant long-term surveillance or limited partial parotidectomy depending on the pathology [22]. Conversely, parotid malignancies require a more aggressive and timely surgical approach [23]. Distinguishing malignant from benign tumors remains challenging because of substantial histologic overlap [24]. Previous studies have reported variable diagnostic accuracy, with the sensitivity of FNAC for detecting parotid malignancy ranging from 59% to 93.5%, compared with 81.1% to 96.7% for CNB [25]. To standardize the reporting of salivary gland cytology, the Milan System for Reporting Salivary Gland Cytopathology (MSRSGC) was introduced in 2018 [26,27]. This system assigns each diagnostic category a specific risk of malignancy, thereby improving communication between pathologists and clinicians, reducing unnecessary surgeries, and guiding management strategies [28]. Subsequent studies have validated the MSRSGC for effectively stratifying malignancy risk in salivary gland FNAC samples, with refinements in recent editions [29]. Our cohort included patients from 2014 to 2023, encompassing the period before the adoption of the MSRSGC; thus, cytologic results in our study could not be classified according to this system.

Among the patients who underwent pathologic evaluation, the proportion of head and neck cancers was relatively high, likely because head and neck surgeons are more vigilant in assessing parotid lesions in patients with primary malignancies in this region. A recent review of metastatic parotid tumors identified squamous cell carcinoma as the most common primary source, followed by malignant melanoma and Merkel cell carcinoma [30]. Notably, three cases in our study were initially diagnosed as benign on FNAC or CNB but were subsequently confirmed as malignant on final pathology. Therefore, parotidectomy should be considered when the clinical condition allows, to establish a definite diagnosis.

Most patients in our cohort had a short follow-up period after detection of incidental parotid lesions, primarily due to severe underlying conditions or loss to follow-up. Among those who were observed without parotid surgery, none developed parotid-related complications. As is well known, when general condition permits, parotidectomy serves as both a diagnostic and therapeutic procedure. However, in patients with poor overall health, additional evaluation should be prioritized when the primary malignancy is located in the head and neck region, particularly in cases of skin cancer, to rule out possible parotid metastasis.

Our study has several limitations. First, the number of patients who underwent further evaluation for parotid incidentalomas was limited, resulting in a small subset with pathologic confirmation. This likely reflects the characteristics of our cohort—approximately half of the PET-CT group had advanced-stage cancers, while many MRI patients presented with acute conditions such as stroke or trauma that required immediate management, precluding further evaluation. In such cases, the decision to pursue surgery requires careful clinical judgement. Second, the retrospective, single-center design introduces potential selection bias. Nevertheless, given the relatively large number of PET-CT and MRI examinations analyzed, the impact of such bias may be minimized. Despite these limitations, our study provides valuable data on clinical outcomes and patients’ general conditions, thereby reflecting real-world clinical practice.

## 5. Conclusions

Many parotid incidentalomas are benign, and treatment decisions should be individualized according to the patient’s overall condition. However, parotid incidentalomas detected on PET-CT often occur in patients with primary malignancies, necessitating careful evaluation to determine whether these lesions represent metastases or a second primary tumor. In particular, in patients with skin cancer or head and neck primary tumors, the possibility of parotid metastasis should be thoroughly investigated.

## Figures and Tables

**Table 1:** General characteristics of patients with incidentally detected parotid abnormalities based on brain MRI and PET-CT interpretations.

Characteristics	Brain MRI	PET-CT
Incidence	100/44,952 (0.22%)	92/10,957 (0.84%)
Sex (Male:Female)	47:53	68:24
Age (years)	68.90 ± 12.61	69.49 ± 11.64
Initial pathologic evaluation (FNAC ^1^ or CNB ^2^)	35/100 (35.0%)	23/92 (25.0%)

^1^ FNAC; fine-needle aspiration cytology. ^2^ CNB; core-needle biopsy.

**Table 2:** Indications for PET-CT imaging of parotid incidentalomas.

Indication	Cases (*n*)	Stage I−II	Stage III−IV
Gastric cancer	5	2	3
Lung cancer	28	8	20
Sarcoma	1	0	1
Hepato-biliary cancer	6	3	3
Breast cancer	1	1	0
Colorectal cancer	11	9	2
Primary malignancy of unknown origin	3	0	1
Esophageal cancer	3	1	2
Head and Neck cancer	11	9	2
Ovary/Cervix cancer	5	2	3
Skin cancer	6	3	3
Pancreas cancer	1	0	1
Prostate cancer	4	2	2
Thyroid cancer	6	6	0
Neuroblastoma	1		
Screening of health	1		
Total	92	47	45

**Table 3:** Patient characteristics according to whether further evaluation (e.g., fine needle aspiration cytology or core needle biopsy) was performed among patients with incidentally detected parotid lesions on brain MRI.

Characteristics	Further Evaluation				*p*-Value	
	Performed (*n* = 35)		Not Performed (*n* = 65)			
Sex (Male:Female)	15:20		32:33		0.675	
Age (years)	69.20 ± 10.23		68.74 ± 13.80		0.182	
Size (cm)	1.87 ± 0.61		1.83 ± 0.99		0.067	
ECOG (0–1:2–4)	27:8		48:17		0.811	
ASA (1–2:3–4)	12:23		27:38		0.312	
Indication	Cancer work-up Brain tumor Dementia Stroke Trauma/Hemorrhage Aneurysm Neuropathy Dizziness Infection/inflammation Seizure Headache	2 2 4 14 2 2 7 2 0 0 0	Cancer work-up Brain tumor Dementia Stroke Trauma/Hemorrhage Aneurysm Neuropathy Dizziness Infection/inflammation Seizure Headache	9 2 4 23 2 1 4 8 3 5 4		

**Table 4:** Patient characteristics according to whether further evaluation (e.g., fine needle aspiration cytology or core needle biopsy) was performed among patients with incidentally detected parotid lesions on PET-CT.

Characteristics	Further Evaluation				*p*-Value
	Performed (*n* = 23)		Not Performed (*n* = 69)		
Sex (Male:Female)	16:7		51:18		0.331
Age (years)	68.70 ± 13.29		69.52 ± 11.31		0.772
SUV	7.02 ± 2.97		6.77 ± 2.73		0.859
Stage (I–II:III–IV)	17:6		30:39		0.016
Primary site	Colorectal Esophageal Hepatobiliary Head and Neck Lung Ovary/Cervix Sarcoma Skin Thyroid	3 2 1 8 3 1 1 3 2	Breast Colorectal MUO Esophageal Gastric Hepatobiliary Head and Neck Lung Ovary/Cervix Pancreas Prostate Skin Thyroid	1 8 3 1 5 5 3 25 4 1 4 3 4	

MUO; malignancy of unknown origin.

## Data Availability

The original contributions presented in this study are included in the article. Further inquiries can be directed to the corresponding author.
